# Recurrent Hypoglycemia Secondary to Insulinoma in an Adult With Beckwith-Wiedemann Syndrome

**DOI:** 10.1210/jcemcr/luad062

**Published:** 2023-06-28

**Authors:** Tugce Akcan, Julia Rose R Shariff

**Affiliations:** Internal Medicine, Marshfield Clinic Health System, Marshfield 54449, USA; Endocrinology, Diabetes and Metabolism, University of Wisconsin School of Medicine and Public Health, Madison 53792, USA

**Keywords:** hypoglycemia, hyperinsulinemia, insulinoma, beckwith-wiedemann syndrome, heart failure

## Abstract

Beckwith-Wiedemann syndrome (BWS) is a rare genetic disorder characterized by genetic and epigenetic changes on the chromosome 11p15.5 region, which includes genes that are important for fetal and postnatal growth. Children with BWS have a higher chance of having hypoglycemia, hyperinsulinemia, and malignancies early in life, although hypoglycemia caused by an insulinoma that develops later in life has not been reported. We describe the diagnosis of insulinoma in a 53-year-old man with BWS in this case report. This is the first case report of insulinoma in an adult with this syndrome.

## Introduction

Beckwith-Wiedemann syndrome (BWS) is a genetic disorder involving macrosomia, macroglossia, hemihypertrophy, childhood tumors, and neonatal hypoglycemia and hyperinsulinemia. The etiology behind this hyperinsulinemic hypoglycemia is not well understood but is thought to be due to chromosomal mutations at 11p15.5 and genes encoding β-cell ATP-sensitive potassium channels [1]. In our case, we present an adult patient with a history of BWS presenting with hypoglycemia from another cause: an insulinoma. Insulinomas are neuroendocrine tumors involving pancreatic cells from the ductal and acinar system [2]. The overproduction of insulin leads to recurrent and unpredictable hypoglycemia. It has been proposed that IGF-2 pathway genes are upregulated in insulinomas, which may also play a role in BWS tumor predisposition [3-5]. Hypoglycemia caused by an insulinoma that develops later in life in a patient with BWS, on the other hand, has not been reported.

## Case Presentation

The patient is a 53-year-old male with a medical history of BWS. He was admitted because of shortness of breath and was found to have new and severe systolic heart failure. Ejection fraction at that time was approximately 25% and not found to be ischemic in nature. He was started on β blockers and evaluated by cardiology. During his hospital stay, he developed hypoglycemia with glucose levels in the 20s. He was started on dextrose because the hypoglycemia became recurrent. Dextrose was shut off to obtain hypoglycemia laboratory values including insulin, proinsulin, C-peptide, and β hydroxybutyrate; however, his blood sugar never dropped below 80 mg/dL (4.4 mmol/L). His hypoglycemia was attributed to a side effect of the β blocker or rebound from dextrose infusion. He was discharged home at the time.

Two weeks later, the patient was readmitted after a loss-of-consciousness episode in his home. He was putting the dishes away in his kitchen when, without warning, he lost consciousness. His blood glucose was 18 mg/dL (1 mmol/L) on arrival to the emergency department; he was given dextrose.

## Diagnostic Assessment

**Figure 1.**
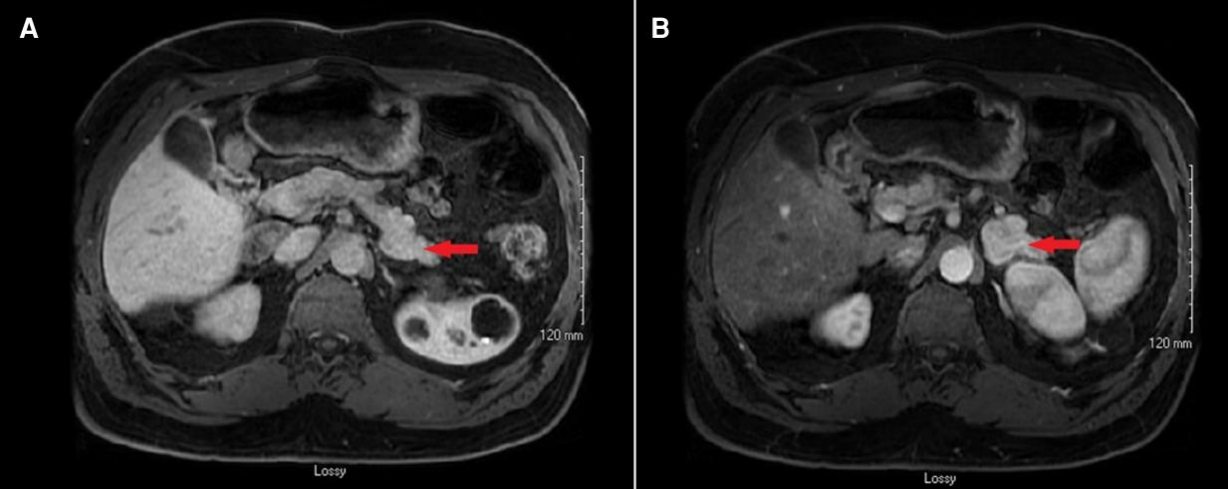
(A) Computed tomography image of the abdomen, demonstrating a large heterogeneous enhancing insulinoma arising from the tail of the pancreas. (B) Magnetic resonance imaging scan of the abdomen revealing of a mass in the tail of the pancreas, which represents neuroendocrine tumor/insulinoma.

Dextrose was again stopped after admission to the hospital and his blood glucose dropped to 45 mg/dL (2.5 mmol/L) on serum testing. At that time, C peptide was elevated at 25.7 ng/mL (8.55 nmol/L), β hydroxybutyrate was 0.62 mg/dL (0.06 mmol/L), and insulin levels were >700 uIU/mL (>4861.5 pmol/L). The differential diagnosis at that time was between insulin autoantibody syndrome or insulinoma. The patient then underwent computed tomography scanning of the abdomen that demonstrated a neuroendocrine tumor measuring 3.7 × 2.9 cm in the distal pancreas (Fig. 1A). Changes in the liver were observed that were concerning for metastases, therefore a magnetic resonance imaging was ordered to reassess this. The magnetic resonance imaging scan did not demonstrate evidence of liver metastasis (Fig. 1B).

## Treatment

**Figure 2.**
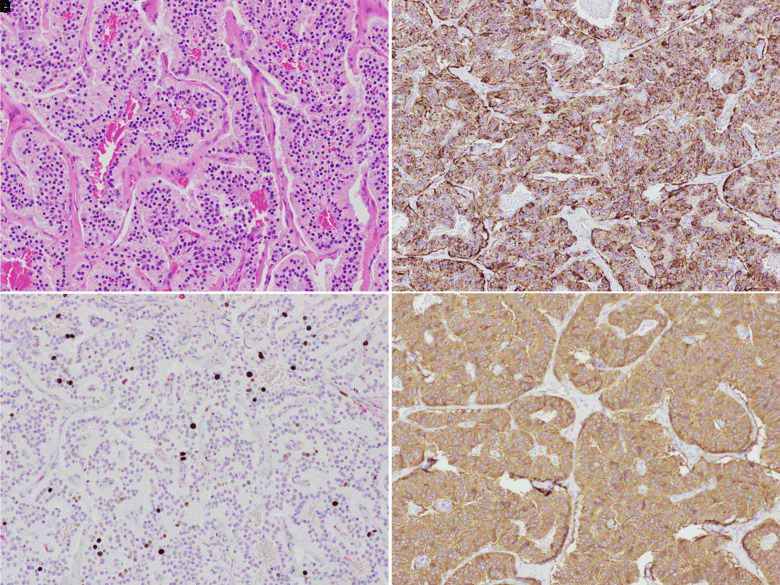
Histological sections of the insulinoma. (A) Histopathological appearance of insulinoma, hematoxylin and eosin. (B) Immunohistochemistry staining showing positivity of tumor cells for chromogranin. (C). Immunoperoxidase stain for Ki-67. (D) Immunohistochemical reaction for chromogranin.

In the meantime, the patient was started on octreotide 50 mcg subcutaneously twice daily. Diazoxide was not pursued given patient’s underlying heart failure. Over the course of his hospital stay, the patient’s octreotide dosage was increased to 100 mcg subcutaneously 3 times daily and discharged home with a continuous glucose monitor with plans to operate later that month. The patient underwent surgery for management of a presumed insulinoma. Surgical pathology revealed a T2 lesion with 2 positive nodes, demonstrating a well-differentiated pancreatic neuroendocrine tumor. Cells were positive for chromogranin and synaptophysin, and Ki-67 staining was 3.5%, supporting the diagnosis of pancreatic neuroendocrine tumor (Figure 2).

## Outcomes and Follow-up

The patient's hypoglycemia resolved postoperatively, and he was taken off octreotide completely without further hypoglycemia.

Notably, before surgery and while being successfully treated with octreotide the patient underwent a repeat echocardiogram, which revealed a significant improvement from 25% to 55% left ventricular ejection fraction.

## Discussion

A patient with BWS developing insulinoma at the age of 53 years is one of this case's distinguishing features. Although hypoglycemia induced by an insulinoma that develops later in life has not been recorded, children with BWS are known to experience hypoglycemia, hyperinsulinemia, and malignancies early in life. BWS is an overgrowth syndrome and cancer predisposition disorder [1]. BWS is caused by defects of the imprinting centers in the chromosome 11p15.5 region and is characterized by a wide spectrum of symptoms and physical findings [5]. It is also associated with hyperinsulinemic hypoglycemia, which affects approximately 50% of children with BWS, and was found to be caused by the loss of functional KATP channels in pancreatic cells [1]. It has been proposed that tumor predisposition in BWS is related to the imprinting status of genes on chromosome 11p15.5, and that one of those genes, IGF2, plays an important role in childhood overgrowth syndromes as well as the development of insulinoma, which could explain our case [3-6]. Given this is such a rare disorder, there is a paucity of information on predisposition or risk factors. There are no recorded cases of insulinoma causing hypoglycemia in a patient with BWS, nor is there any evidence that it could be a risk factor for this syndrome. However, the link between macrosomia and tumor growth in BWS suggests that there may be a predisposition to insulinoma that has yet to be investigated. Another distinguishing feature of our case is that it began with heart failure, which significantly improved with insulinoma treatment. Although the pathophysiological connection is not definitively established, insulinoma has been linked to cardiomyopathy in some cases [7]. One proposed mechanism is that hyperinsulinemia increases cellular ATP levels through increased glucose metabolism, which then closes cardiac KATP channels, which have been linked to cardiomyopathy [8,9]. Another proposed mechanism is hypoglycemia causing catecholamine excess, which has previously been observed in patients with anorexia nervosa [10]. Our patient's initial heart failure presentation may have been influenced by repeated hypoglycemia and hyperinsulinemia caused by an underlying insulinoma.

## Learning Points

- It is unclear whether Beckwith-Wiedemann syndrome (BWS) could be a predisposing factor to the development of insulinoma given predilection to childhood tumors associated with BWS.
- The link between macrosomia and tumor growth in BWS suggests that there may be a predisposition to insulinoma that has yet to be investigated.
- The patient's initial heart failure presentation may have been affected by recurrent hypoglycemia and hyperinsulinemia. Although the pathophysiological link has not been shown, insulinoma has been associated to cardiomyopathy in rare cases.

## Contributors

Individual contributions to authorship were made by all authors. J.R.S. was engaged in the patient's diagnosis and management. The manuscript was written, reviewed, and edited by T. A. and J.R.S. The final manuscript was reviewed and approved by both authors.

## Data Availability

All data are presented in the manuscript.

## Abbreviation

BWS: Beckwith-Wiedemann syndrome
